# Familial Resemblance of Bone Health in Maternal Lineage Pairs and Triads: A Scoping Review

**DOI:** 10.3390/life14070819

**Published:** 2024-06-27

**Authors:** Nicole M. J. Boisvert, Melissa R. McElroy, K. Alix Hayden, Patricia K. Doyle-Baker

**Affiliations:** 1 Human Performance Lab, Faculty of Kinesiology, University of Calgary, Calgary, AB T2N 1N4, Canada; 2 Northern Ontario School of Medicine University, Sudbury, ON P3E 5Z6, Canada; mmcelroy@nosm.ca; 3 Libraries and Cultural Resources, University of Calgary, Calgary, AB T2N 1N4, Canada; ahayden@uclagary.ca; 4 Alberta Children’s Hospital Research Institute, University of Calgary, Calgary, AB T2N 1N4, Canada

**Keywords:** bone, bone mineral density, bone mass, bone health, familial resemblance, heritability, women’s health, mothers, daughters

## Abstract

Introduction: Female bone health is influenced by familial resemblance, health parameters and maturational periods (puberty and menopause); this combination has been researched using familial multi-generational cross-sectional studies. Aim: This scoping review aimed to compile bone health research which uses sexually mature (grandmother–) mother–daughter pairs (and triads) and to determine the trends in its methodologies and familial comparisons. Methods: The Joanna Briggs Institute methodology for scoping reviews was used. Extraction included study and population characteristics, methodology (with an emphasis on imaging) and family-based results. Results: Twenty-nine studies were included, and their generations were categorized into four developmental categories: late adolescent to young adult, pre-menopause, mixed-menopause, and post-menopause. Eleven different pair/triad combinations were observed; the most common was pre-menopausal daughters and post-menopausal mothers. Dual-energy X-ray absorptiometry (DXA) was the most utilized imaging modality, and the hip was the most imaged region of interest (ROI). Regardless of pairing, imaging modality and ROI, there was often a trend toward significant familial resemblance and heritability (h^2^ and h^2^_L_). Conclusion: This scoping review highlights the trends in bone health linked to familial resemblance, as well as the importance of menopause and late adolescence. This review compiles the commonalities and challenges within these studies to inform future research.

## 1. Introduction

Osteoporosis, a systemic skeletal disorder of low bone mass [1] results in a decreased quality of life [2] and an increased risk of fragility and fracture [1]. Moreover, if an individual with osteoporosis does fracture a bone, their quality of life will be further reduced [3,4], and their risk of another fracture is increased [5], as is death [6]. Females make up the majority of those diagnosed with osteoporosis, with nearly six osteoporotic females for every osteoporotic male in Canada [7]. Being female, in and of itself, is a risk factor for osteoporosis; they also experience the majority of osteoporotic fractures [8]. There are also several female sex-specific risk factors, such as menopause [9], a time during which there is a substantial acceleration in bone loss [10].

In contrast to this female-specific accelerated decline, there is a period during the peripubertal years where a substantial amount of bone accrual is experienced. Bone mass continues to accrue until a plateau is achieved in early adulthood; this is called peak bone mass (PBM) [11,12]. Like how menopause-related bone loss affects only female-sexed persons, there is a difference between the sexes during this period of heightened accrual. Females’ bone mass plateau is earlier than males, resulting in them having less time to accrue mass; subsequently, they achieve a lower overall PBM [13]. Optimizing the PBM is critical, as it is a predictor of the future risk of osteoporosis and fragility fractures [9,14]. Therefore, both menopause and young adulthood are important life stages for studying bone health and for understanding osteoporosis and its outcomes in females. 

A longitudinal design would be ideal for studying bone health at these key life stages. However, to circumvent the high cost and participant burden that accompany such studies, many researchers have used a specific cross-sectional design. This design consists of parent–offspring pairs or grandparent–parent–offspring triads. Bone variance is highly (60–80%) inherited [11,15], thus these familial pairs and triads are used to answer similar questions to those that may be investigated through a longitudinal study.

The purpose of this scoping review was to compile the existing grandmother–mother–daughter and mother–daughter cross-sectional studies to examine the statistically determined relationships and differences in their bone health parameters. We specifically wanted to identify and understand the familial resemblances in bone health within maternal lineage pairs and triads. To do this we investigated how mother–daughter bone health studies were conducted and how the literature reported the comparisons and relationships in bone health measures between maternal triads and pairs. Our overall goal was to synthesize the current knowledge on maternal triads or pairs in terms of familial resemblance as it relates to bone health parameters. 

## 2. Materials and Methods

The PCC (population, concept, context) framework [16] was used to describe the inclusion and exclusion criteria (Table 1).

This review followed the Joanna Briggs Institute (JBI) methodology for scoping reviews [16] and is reported per the Preferred Reporting Items for Systematic Reviews and Meta-Analyses extension for Scoping Reviews (PRISMA-ScR) [17] (Table S1). The JBI guidance for scoping reviews outlines a three-stage process as the search strategy. An initial discovery search was conducted to find relevant studies to guide the development of the search strategy. Content experts on the research team provided key studies, Google Scholar was searched, and these studies were analyzed for text words in their titles, abstracts and keywords. This was followed by a draft Medline search to test whether the key studies were retrieved. The final search strategy focused on two main concepts: mother–daughter–grandmother triads/pairs and bone density. Both keywords and subject headings were used for each concept. Keywords were standard across all databases, and the subject headings were determined by the controlled vocabulary of the database. No date, country or language limits were applied.

The preliminary protocol was established a priori and registered on 5 May 2021 with the University of Calgary PRISM Kinesiology Research & Publications Collections [18]. After the search was conducted using the preliminary protocol [18], inclusion/exclusion was determined to be too broad to answer the objectives and was narrowed in scope. The menstrual criteria were clarified to include all those who had previously passed menarche as opposed to only the youngest member of mother–daughter pairs to have begun menarche. As opposed to the group of “grandmothers” having to be post-menopausal, the menopausal status of age-relevant groups was required to be accounted for in either the study design (e.g., through grouping) or within the analysis (e.g., adjusting for menopausal status). The quantification of participant bone health was narrowed because the Fracture Risk Assessment Tool (FRAX) score and fracture history alone did not provide enough information to make comparisons between studies. Only those studies that collected imaging-based measurements of bone parameters, such as DXA, single photon absorptiometry (SPA), peripheral quantitative computed tomography (pQCT) or other techniques, were included. Lastly, the definition of familial resemblance was expanded to exclude articles that did not analyze the relationship between offspring and parents’ bones. Thus, only records which utilized a statistical test (e.g., correlations, regression, *t*-tests), to compare pairs or triads were included.

### 2.1. Search Strategy 

**Figure 1:**
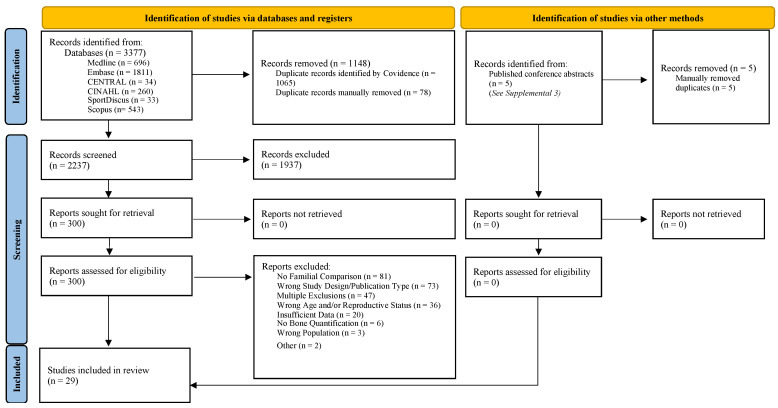
PRISMA diagram following scoping review extension [17,24]. Note: “Wrong Age and/or Reproductive Status” includes studies which did not determine/account for reproductive status (menarche or menopause). “Insufficient data” included mostly conference abstracts for which the full article had already been found or none could be found. From: Page MJ et al. [24].

The original search was conducted between 6 and 9 May in 2021 and a repeat search found no further articles up to April 2024. The databases searched included: MEDLINE(R) and Epub Ahead of Print, In-Process, In-Data-Review & Other Non-Indexed Citations and Daily (OVID), Embase (OVID), the Cochrane Central Register of Controlled Trials (OVID), CINAHL Plus with Full Text (Ebsco), SportDiscus with Full Text (Ebsco), and Scopus (Elsevier). (See Tables S2–S7 for database searches.) The grey literature search included published conference abstracts from seventeen bone-health-related conferences for the years 2010–2021 (see Table S8); however, the five abstracts found were determined to have been duplicates (Figure 1).

### 2.2. Study Selection

Before study selection, a calibration exercise was completed with two reviewers (NB and MM). Their pilot review of 50 records achieved an agreement rate of 96.00%. All identified records were exported and uploaded into Covidence (www.covidence.org; accessed on 6 May 2021) and were auto-de-duplicated. Any records identified as duplicates during screening were removed manually. Titles and abstracts were screened regarding the selection criteria. At this stage, any record without an abstract was not excluded, to avoid rejecting useful records. An agreement rate of 96.66% (Cohen’s kappa = 84.76%) was achieved. Disagreements were discussed and the inclusion/exclusion criteria were clarified when required. Once the full texts of the remaining records were collected, all non-English texts were translated using Google Translate [19]. Full-text records were examined independently by both reviewers with respect to the inclusion/exclusion criteria. The appropriate exclusion reason was assigned by each reviewer, and any differences in inclusion/exclusion were discussed and resolved by NB and MM. The proportion of agreement was 98.08% (Cohen’s kappa = 91.40%). 

### 2.3. Charting the Data

A draft charting table was created prior to extraction, with input from both reviewers, but it was later clarified and expanded upon by the reviewer conducting the extraction. As such, extraction was iterative, both flexible and comprehensive. Details regarding the studies’ characteristics, population, concept, review questions, and key findings were extracted. The extraction of information about bone health parameters, as a concept of interest, included imaging/quantification modalities and the bone site(s) measured. The country extracted was that of the study population, not that of the authors or publication location, due to the link between bone and socioeconomics and race [9,14]. The participant groups/categories included were late adolescent/young adult, pre-menopausal, post-menopausal, and mixed-menopausal. The definitions are presented in Table 2. 

**Table 2:** Category and reproductive status-based definitions.

Category	Definitions
Late adolescent/young adult	The group who had menstruated and had a mean age of <25
Pre-menopausal	The group with a mean age ≥ 25 yrs, whose bone growth was largely finished [12,13] and who were not menopausal
Post-menopausal	The group wherein the women no longer menstruated
Mixed-menopausal	A group of both pre- and post-menopausal women

Menopause was chosen as a cut-off point due to the accelerated bone loss that occurs during and post-menopause [10]. The two pre-menopausal groups were divided according to their PBM. Bone accrual peaks (or plateaus) at some bone sites in a female’s third decade of life [13]. Yet, it has also been shown that accrual is over 95% complete at multiple sites by an earlier age (17.8 ± 1.0 yrs.) and has started decreasing by the age of 25 [12]. 

Bone imaging sites from various modalities were grouped into ROIs based on their common DXA terminology, where possible. For example, the high-resolution pQCT (HR-pQCT) radial site falls within DXA’s ultra-distal radial region (UDRad) [20], whereas the pQCT site is ~1/3 up from the wrist, which is part of DXA’s mid-radius (MRad) ROI (see Figure S1). Bone imaging was then categorized into body segments (ROI category), which included the whole body, forearm (radius and ulna), radius, lumbar spine, hip, femur, tibia and heel. The groupings can be found in Table S9. 

Each study site result was extracted as an individual result because a study may include measures from multiple sites within one ROI category. For example, one study may include two DXA and one HR-pQCT radial ROIs, such as the areal bone mineral density (aBMD) of the UDRad and MRad (1/3 distal radius) from DXA and volumetric bone mineral density (vBMD) from HR-pQCT within the UDRad ROI. In addition to ROI, ROI category and bone health parameters (e.g., aBMD, vBMD, BMC: bone mineral content) were initially extracted for mother–daughter bone comparisons as a statistical test type, result value and significance value. These were then coded as N (non-significant), Y (significant) or UN (unknown significance; lacking p-value or confidence interval). A ratio (Y:N:UN) of the results for each condition (mother–daughter category per ROI) was compiled. The results were grouped by statistical test into (a) similarity (e.g., regression and correlation), (b) difference (e.g., *t*-tests and ANOVAs) and (c) heritability. 

### 2.4. Ratios of Statistical Significance Results 

To decrease the inflation or skewing of ratios, not all bone variables extracted were included in the reported ratios. If the imaging from one modality at one ROI included multiple measures, bone mineral density (aBMD or vBMD) was preferable to BMC, bone mass (BM) or calculated apparent bone mineral density (BMAD). For HR-pQCT and pQCT studies, the total volumetric BMD (Tt.vBMD) was selected over segmented ones (cortical: Ct.vBMD or trabecular: Tb.vBMD). In the case that the Tt.vBMD was not reported, Ct.vBMD was chosen because Ct.vBMD is a higher proportion of the Tt.vBMD than Tb. For studies including both a bone mineral measure (e.g., aBMD) and the corresponding Z-score (e.g., aBMD Z-score), the bone mineral measure was utilized, as fewer studies reported measures as Z-scores. Of the measures taken from quantitative ultrasound (QUS), broadband ultrasound attenuation (BUA) was preferable to the speed of sound (SOS). Both correlate well with aBMD values [21]; however, not all included QUS studies reported both parameters. They did all report BUA. 

Beyond the units of measure, decisions were made regarding the breadth of the scan ROI. In the case of lumbar spine (LS) results, total LS (L1–4) was selected over L2–4, if both were reported. The study population was used to determine the included results when there were controls and case pairs. If results for all pairs were reported, these were chosen. If not, it was the control pairs. However, for Hansen, Hassager [22], there were two distinct sub-populations (1 and 2), and their results were included separately as if they were two unique studies. The mothers in population 2 only had 2/5 ROIs imaged. Population 1 mothers were recruited from a previous study and had historic measures. These were not included in the ratios, as they were of only 1/5 ROIs and they were over a decade earlier than their daughters’ measures. Correlation and regression analyses, statistical tests regarding similarity, were both run in numerous studies. When this happened, the most adjusted value was included in the results’ ratios.

## 3. Results

The initial search identified 3377 records; 1148 duplicate records were removed, 1937 were deemed irrelevant and the remaining 296 records, with full texts, were assessed for their eligibility and 268 were excluded (see Figure 1; PRISMA diagram).

After screening for inclusion/exclusion criteria, the remaining 29 records were included (Table 3). The most common reason for exclusion was a lack of familial bone comparison (81 records). See Table 3 for a list of included studies. There was an erratum discovered in one of the included papers [23]. It corrected the published order of authors for Blain et al. [23] only and was deemed a duplicate.

**Table 3:** Characteristics of the studies by author, year, country and pairing category by participant (number (n), age mean and SD).

Study and Sub-Population	Full Pairing and Triad Numbers		Daughters		Mothers		Grandmothers	
Author, Year, Country	Pairs (Triads)	Pairing Category	n	Age (yrs) Mean ± SD (Range)	n	Age (yrs) Mean ± SD (Range)	n	Age (yrs) Mean ± SD (Range)
Blain et al., 2006 [23], France	86 ^a^	Mixed-Menopausal Daughters and Mothers	90	35.5 ± 13.1 (18–64)	82	62.9 ± 12.8 (38–90)		
Cheng et al., 2009 [25], Finland	144	Adolescent Daughters and Pre-Menopausal Mothers	144	18.3 (18–20)	144	45.0 (32–54)		
Danielson et al., 1999 [26], USA	207	Mixed-Menopausal Daughters and Post-Menopausal Mothers	207	48.5 ± 7.3 (30–70)	207	71.7 ± 4.6 (>65)		
Drozdzowska and Pluskiewicz, 2001 [27], Poland	48	Pre-Menopausal Daughters and Post-Menopausal Mothers	48	43.2 ± 5.7	48	70.9 ± 5.2		
Ferrari et al., 2006 [28], Switzerland	93	Adolescent Daughters and Pre-Menopausal Mothers	93	16.4 ± 0.5	93	39		
Francois et al., 1999 [29], France	175	Adolescent Daughters and Pre-Menopausal Mothers	175	17.4 ± 2.03 (13.4–23.5)	175	42.6 ± 4.1		
Hansen et al., 1992 [22], Denmark (a) Population 1	101 ^b^	Pre-Menopausal Daughters and Post-Menopausal Mothers	144	35 ± 5	101	62 ± 3 (50 ± 3)		
Hansen et al., 1992 [22], Denmark (b) Population 2	148 ^b^	Pre-Menopausal Daughters and Post-Menopausal Mothers	195	42 ± 5	148	70 ± 1		
Henderson et al., 1995 [30], Australia	115 ^c^	Adolescent Daughters and Mixed-Menopausal Mothers	115	18–18.9	107	46.7 ± 4.3		
Jouanny et al., 1995 [31], France	98 ^a^	Adolescent Daughters and Pre-Menopausal Mothers	98	18.1 ± 2.0	129	41.9 ± 3.6		
Krall and Dawson-Hughes, 1993 [32], USA	40	Pre-Menopausal Daughters and Post-Menopausal Mothers	40	31 ± 6 (21–45)	40	60 ± 6 (43–71)		
Lee et al., 2020 [33], Singapore	10	Pre-Menopausal Daughters and Post-Menopausal Mothers	10	29 ± 5 (22–39)	10	63 ± 2 (60–71)		
Lutz and Tesar, 1990 [34], USA	37	Pre-Menopausal Daughters and Mixed-Menopausal Mothers	37	25 ± 4 (20–34)	37	52 ± 7 (41–68)		
Margarey et al., 1999 [35], Australia	39 ^a^	Adolescent Daughters and Mixed-Menopausal Mothers	39	17–17.9	98	44.6 ± 4.8		
McKay et al., 1994 [36], Canada	24	Pre-Menopausal Daughters and Post-Menopausal Mothers	24	40.0 ± 5.4 (26.3–49.1)	24	67.3 ± 6.7 (57.1–78.9)		
Nabulsi et al., 2013 [37], Lebanon	91 ^b^	Adolescent Daughters and Pre-Menopausal Mothers	91	13.8 ± 9.9	169	40.5 ± 5.2 (30–55)		
Nagy et al., 2013 [38], France (a) Fragility-Fractured Mother Pairs	115 ^b^	Pre-Menopausal Daughters and Post-Menopausal Mothers	115	42.9 ± 7.7	92	72.1 ± 7.8		
Nagy et al., 2013 [38], France (b) Non-Fractured Mother (Control) Pairs	206 ^b^	Pre-Menopausal Daughters and Post-Menopausal Mothers	206	38.7 ± 9.2	179	66.7 ± 8.2		
Nagy et al., 2015 [39], France	210 ^b^	Pre-Menopausal Daughters and Post-Menopausal Mothers	210	38 ± 9	171	65 ± 9		
Ohta et al., 2010 [40], Japan	339 ^DM^ (34 ^MG^)	Full Triads: Adolescent Daughters, Pre-Menopausal Mothers and Post-Menopausal Grandmothers	339	14.8 ± 1.7 (12–18)	339	46.4 ± 4.0	34	77.9 ± 4.5
Pepe et al., 2017 [41], Switzerland	102	Adolescent Daughters and Mixed-Menopausal Mothers	102	20.4 ± 0.5	102	50.6 ± 4.1		
Picard et al., 2001 [42], Canada	70 ^b^	Pre-Menopausal Daughters and Mothers	70	26.6 ± 4.7	58	44.1 ± 2.7		
Runyan et al., 2003 [43], USA	22 ^a^	Pre-Menopausal Daughters and Post-Menopausal Mothers	72	42.4 ± 4.2 (33–51)	22	67.6 ± 8.8 (52–87)		
Seeman et al., 1989 [44], Australia (a) Control Mother Pairs	22 ^b^	Pre-Menopausal Daughters and Post-Menopausal Mothers	22	34.6 ± 1.9	20	62.8 ± 2.3		
Seeman et al., 1989 [44], Australia (b) Osteoporotic Mother Pairs	32 ^b^	Pre-Menopausal Daughters and Post-Menopausal Mothers	32	36.9 ± 1.4	25	67.9 ± 1.5		
Shetty et al., 2016 [45], India	150	Pre-Menopausal Daughters and Post-Menopausal Mothers	150	35.6 ± 5.4	150	59.0 ± 5.4		
Sowers et al., 1986 [46], USA	34 ^b^	Mixed-Menopausal Daughters and Mothers	36	31 ± 9	34	56 ± 10		
Tylvasky et al., 1989 [47], USA	84	Adolescent Daughters and Pre-Menopausal Mothers	84	18.6 ± 0.1 (17–23)	84	44.2 ± 0.4 (35–39)		
Ulrich et al., 1996 [48], USA	25	Pre-Menopausal Daughters and Post-Menopausal Mothers	25	41 ± 5	25	72 ± 5		
Wang et al., 2011 [49], Finland	55 ^DM,MG^ (55)	Full Triads: Adolescent Daughters, Pre-Menopausal Mothers and Post-Menopausal Grandmothers	55	18.1 ± 1.0	55	43.6 ± 3.1	55	68.0 ± 4.4
Wang et al., 2015 [50], Finland	128 ^DM,MG^ (128)	Full Triads: Adolescent Daughters, Pre-Menopausal Mothers and Post-Menopausal Grandmothers	224	18.3 ± 1.1	128	44.9 ± 4.1	128	70.0 ± 6.3
Xu et al., 2010 [51], Finland	100 ^DM a^ (44 ^MG a^) ^a^	Full Triads: Adolescent Daughters, Pre-Menopausal Mothers and Post-Menopausal Grandmothers	235	18.3 ± 1.1	138	44.7± 4.1	114	70.7 ± 6.3

Hansen et al., 1992 [22]: (a) Population 1: pairs with historical data for mothers from 1977 (~12 years prior; mean age 50 ± 3 years). (b) Population 2: pairs with no historical data. ^MG^: mother–grandmother pairs. ^DM^: daughter–mother pairs. ^a^: studies with additional family members not included in this review (e.g., fathers, sons or pre-menarche daughters). ^b^: Studies with multiple daughters per mother. Number of pairs is dependent on how these groups were handled. ^c^: studies where the main analysis was run on daughters, with additional analysis run if mothers were included.

### 3.1. Study Characteristics 

The count of included studies by country, publication year (per 5 years), imaging modality and scan ROI category are described in Table 4. All publications included populations from a single country from one of four continents: North America (n = 9), Europe (n = 13), Asia (n = 4) and Oceania (n = 3). The single country with the greatest number of studies (n = 7) was the United States [26,32,34,43,46,47,48]. 

Four of the included studies [25,49,50,51] utilized data from the greater CALEX dataset [52,53,54]. Two studies [38,39] used the MODAM study dataset, which recruited 78% of its participants from the OFELY dataset [55]. Danielson and Cauley’s study [26] was conducted by recruiting daughters from mothers in the Study of Osteoporosis Fractures (SOF) study [56].

The earliest paper [46] was published in 1986 and the latest [33] was published in 2020. The most commonly used imaging modality was DXA, which appeared in 18 studies [22,23,25,26,28,30,31,32,33,36,37,38,39,41,43,45,48,50], of which six studies used it in concert with at least one other modality (+QUS and SXA [26]; +SPA [22]; +SPA and DPA [32]; +HR-pQCT [38,39,41]). The least common modality was SXA, which was used in one study [26] with DXA and QUS.

The included studies spanned numerous decades and it is important to highlight that imaging technology was advancing at this time. The focus of the studies also shifted across these decades. Earlier studies, including the majority of those published in 1995 and earlier, had the aim of determining the existence and extent of familial resemblance in bone health [22,32,34,36,44,46,47]. Subsequent studies published in the late 1990s and early 2000s expanded upon this literature. They sought to determine the extent to which familial resemblance contributes to bone health, especially during developmental and maturational periods, as well as its relationship with other factors, such as lifestyle [23,25,26,27,28,29,35,36,42,43,48]. Most of the more recent studies have endeavored to fill in gaps in the literature. This included gaps such as ethnic and geographic population-specific differences [40,45] and the interplay with bone turnover and other physiological markers [33,39,45], as well as the heritability difference for within- and across-sex familial pairings [37,41]. Due to the earlier literature determining the fundamentals of bone heritability, the only gaps were those relating to advanced imaging. Heritability was investigated for bone microarchitecture and strength values in several studies [38,41,51]. Similarly, knowledge regarding the contribution of familial resemblance was leveraged to investigate bone-health-affecting factors and bone health trajectories across the participants’ lifespan [37,49,50].

To facilitate comparison, the scanned ROIs were grouped (see Table S9) into eight categories (Table 5). The category with the highest number of regions (n = 5) was the hip (total hip, TH; femoral neck, FN; trochanter, Troch; intertrochanter, iTroch; and Ward’s triangle/area, Ward). The hip was the most imaged ROI category, with 17 studies imaging at least one of its five ROIs [22,23,26,28,30,32,33,34,36,37,38,39,41,43,44,45,50].

### 3.2. Daughter-Mother and Triad Groupings 

**Figure 2:**
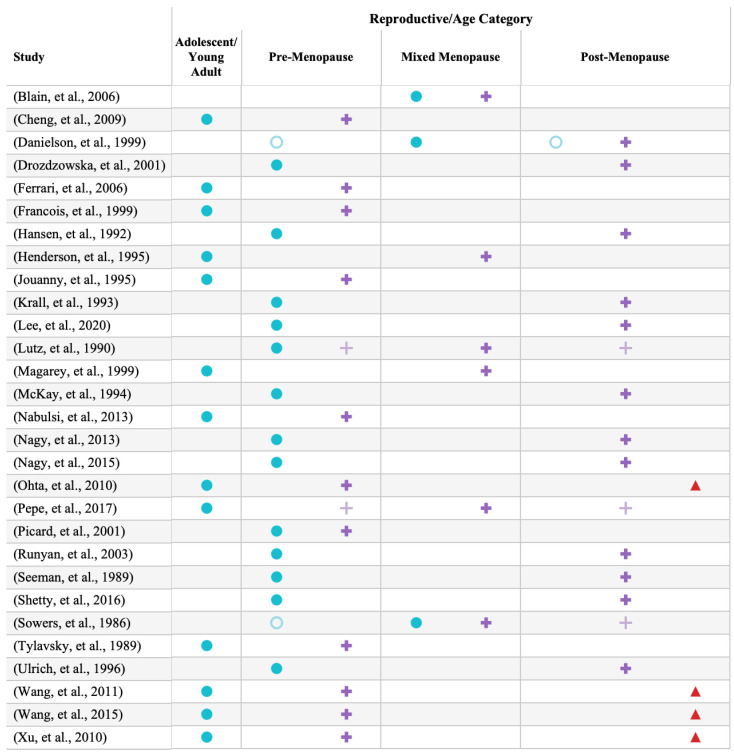
Study-specific representation of age/reproductive category of daughters (blue circles: ●), mothers (purple crosses: +) and grandmothers (red triangles: ▲). Outlined, lighter icons represent generations which were separated based on their menopausal status during their analysis [22,23,25,26,27,28,29,30,31,32,33,34,35,36,37,38,39,40,41,42,43,44,45,46,47,48,49,50,51].

Four studies contained three generational groups [40,49,50,51] consisting of adolescent–young adult-aged daughters, pre-menopausal mothers, and post-menopausal grandmothers. The most frequent pairing was pre-menopausal daughters and post-menopausal mothers [22,27,32,33,36,38,39,43,44,45,48]. Importantly, a number of the studies which included mixed-menopausal daughters or mothers sub-divided the mixed-menopausal group by menopausal status during their analysis. These are indicated in Figure 2 using the outlined versions of the daughter, mother, and grandmother icons. 

### 3.3. Multiple Populations

Several studies included sub-groupings of mother–daughter pairs (see Table 3). Fragility fractures (low trauma fractures, such as from a fall from standing height or less) in mothers defined the “case” pairs in two studies [27,38]. Danielson and Cauley [26] divided pairs into three groups, defined by their maternal bone quality. These groups were the “normal” (control) group; the “low BMD” group, with osteoporotic mothers (a T-score < −2.5); and the “fractured” mothers with osteoporosis (fragility fracture or vertebral deformity). As the T-score used by Danielson and Cauley [26] is the one that the World Health Organization recommends for diagnosing osteoporosis, it was also utilized in two other included studies to define their “case” populations [29,44]. Hansen and colleagues [22] reported two pair populations (as discussed previously); the first had additional historical measures for the mother from a previous study and the second had fewer scan ROIs for the mothers. 

### 3.4. Multiple Daughters

Some studies included more than one daughter per mother. The potential additive effect this could have on their analysis was handled in a few distinct ways. The simplest way was by calculating the arithmetic mean of the daughters to use in mother–daughter statistical tests [22,44]. Another method used was hierarchical models, in which sisters were clustered under the mother [37,42]. The two MODAM studies utilize the same intraclass correlation (ICC) method; with one [39] referencing the methodology of the other [38]. Sowers and Burns [46] utilize a method to adjust significance, which was described by Rosner and Donner [57]. 

Although not discussed within this review, several of the included studies contained additional family members. Both fathers and sons, as mother–father–daughter–son families and in various other combinations, were found in four studies [31,32,33,35]. Mother and son pairings were observed alongside mother–daughter pairs by McKay and Bailey [36], Nabulsi and Mahfoud [37] and Pepe and Biver [41]. Sowers and Burns [46] had a variety of female family groups (which included siblings, aunts, nieces and cousins). Seeman and Hopper [44], which was the eldest study, did not have additional family members, but did include pre- and post-menopausal reference populations for comparison.

### 3.5. Secondary Outcomes: Mother–Daughter Bone Health Results 

Within each of the following statistical and pair groupings, the ratios for ROI categories containing multiple ROIs are referenced by ROI. An example could be a 4:2 significant to non-significant ratio at the hip, with three significant studies across four sites and two ROIs showing non-significance within one study (e.g., TH [study 1 reference, study 2 reference], FN [study 1 reference], and Troch [study 3 reference]: TH [study 3 reference] and FN [study 3 reference]). The ratios and their references can be found in Tables S10–S12.

#### 3.5.1. Familial Bone Differences 

**Figure 3:**
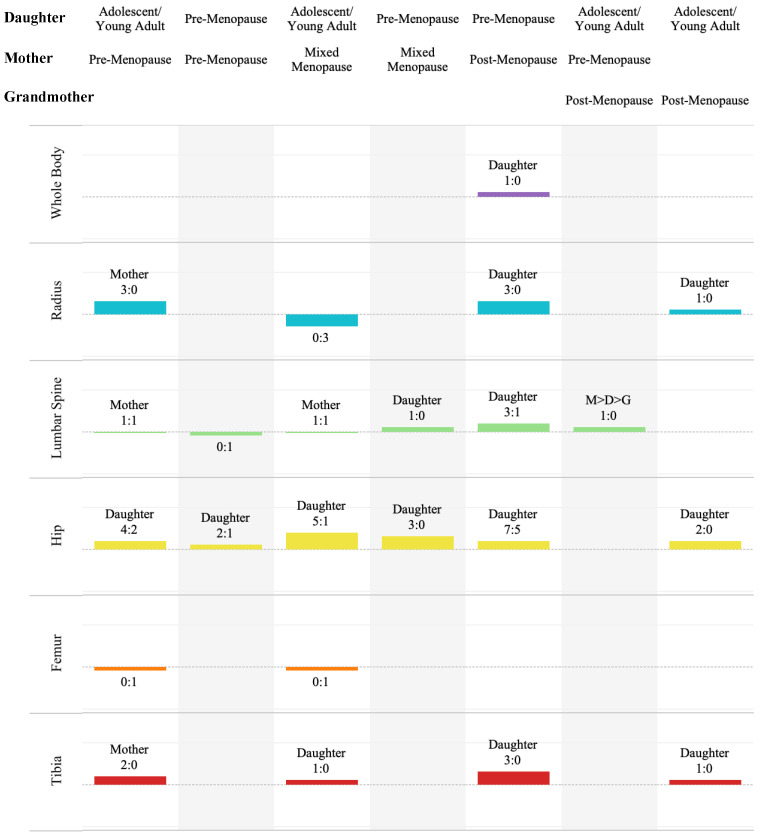
The sum and ratio (sig:non) of significant (+1) and non-significant (−1) differences by daughter–mother pairing and ROI category, with the labelled generation being the one which was more commonly reported as having the greater value.

Ratios of the significant and non-significant statistical differences between bone parameters for grandmothers, mothers and daughters (e.g., *t*-test and ANOVAs) can be seen in Figure 3 (and Table S10). Amongst the included studies, there were no applicable (to this scoping review) results for mixed-menopausal daughters and mothers, mixed-menopausal daughters and post-menopausal mothers, post-menopausal daughters and mothers, or adolescent daughters and post-menopausal grandmothers. Forearm and heel ROIs also lacked a statistical analysis of their differences. 

##### Adolescent/Young Adult-Aged Daughters and Pre-Menopausal Mothers

Most results within this paring displayed a significant difference in bone quality. Two radial results (MDRad and MRad [47]) showed a significant difference and one did not (MRad [49]). The hip results had a ratio of 4:2 (TH [30], Troch [30], and FN [30,50]: TH [50], iTroch [30]). Only Henderson and Price [30] reported femoral results, which were not significant. Both studies measuring the proximal tibia found significant differences [49,51]. 

##### Pre-Menopausal Daughters and Mothers

Lutz and Tesar [34] were the only study to report on differences in this pairing. Their L2–4 aBMD was non-significant. Of the hip ROIs, significant differences were seen only at the trochanter, and not the FN or Ward’s triangle.

##### Adolescent/Young Adult-Aged Daughters and Mixed-Menopausal Mothers

No significant differences were seen at the femur [30], nor in the radial vBMD (UDRad [41]) or aBMD (UDRad and MRad [41]). The lumbar spine results were significant in one study [30], but not in another [41]. The one and only tibial result was significant [41]. The hip ratio was 5:1 (TH [30,41], FN [30,41] and Troch [30]: iTroch [30]). 

##### Pre-Menopausal Daughters and Mixed-Menopausal Mothers

Significant differences in aBMD were observed at all sites (LS and Hip: FN, Troch and Ward) by Lutz and Tesar [34]. 

##### Pre-Menopausal Daughters and Post-Menopausal Mothers

Significant differences were seen for the whole body aBMD [48], radial aBMD (UDRad [39]) and vBMD (UDRad [39] and MRad [49]) and proximal tibia vBMD [39,49,51]. In the LS, three results were significant (LS [33,45] and L2–4 [34]) and one was not (LS [36]). The 7:5 hip results broken down by ROI include the TH at 2:2 ([39,50]:[33,36]); FN at 4:1 ([33,34,45,50]:[39]); Troch at 0:2 [34,36]; and Ward’s at 1:0 [34].

##### Adolescent/Young Adult Daughters, Pre-Menopausal Mothers and Post-Menopausal Grandmothers

Ohta and Kuroda [40] reported a difference in L2–4 aBMD within their triads.

##### Adolescent/Young Adult-Aged Daughters and Post-Menopausal Grandmothers

Daughters and grandmothers were significantly different at the mid-radius and proximal tibia [49] and the hip (TH and FN [39]).

#### 3.5.2. Familial Bone Regressions/Correlations 

**Figure 4:**
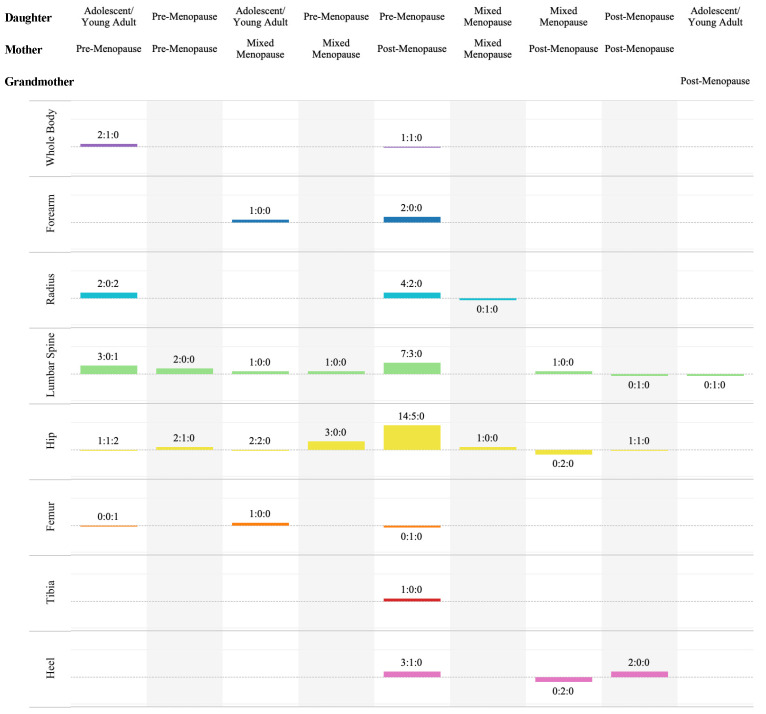
Sum and ratio (sig:non:unknown) of significant (+1) and non-significant (−1) regression/correlation results by daughter–mother pairing and ROI category.

Ratios of the significant and non-significant statistical relationships (e.g., from correlations and regressions) can be seen in Figure 4 (and Table S11). As noted previously, in studies wherein there were multiple values for the same site (by the same imaging technique), the most adjusted value was included in these ratios. There were no correlation or regression results for triads or adolescent daughters and post-menopausal mothers within the included studies.

##### Adolescent/Young Adult-Aged Daughters and Pre-Menopausal Mothers

Ferrari and Chevalley [28] were the only paper to report femoral comparisons; however, they did not provide a p-value or confidence interval for any of their ROIs (Fem, Rad: UDRad and MRad, L2–4, hip: FN and Troch). Thus, the trends in the significance of regression and correlation within this pairing at the femur are unknown. The whole body, with a ratio of 2:1 ([25,31]:[37]), was primarily significant. At the radius, there were two significant ROI results (MDRad and MRad [47]) and two unknowns (UDRad and MRad [28]). Three lumbar spine results (LS [37] and L2–4 [29,40]) showed significance. The hip ratio was 1:1 (TH [37]: FN [37]).

##### Pre-Menopausal Daughters and Mother

The lumbar spine (L2–4 [34,42]) and hip categories (FN and Ward [34]) each include two significant results. However, another hip result was not significant (Troch [34]). 

##### Adolescent/Young Adult-Aged Daughters and Mixed-Menopausal Mothers

Within this pairing, there was only one ROI result for the forearm [35], lumbar spine (LS [30]), and femur [30] categories, all of which were significant. Henderson, Price [30] studied four hip ROIs; two showed significance (FN and Troch) and two did not (TH and iTroch). 

##### Pre-Menopausal Daughters and Mixed-Menopausal Mothers

A significant relationship in this pairings’ aBMD was seen at the hip (FN, Troch and Ward) and lumbar spine by Lutz and Tesar [34]. 

##### Mixed-Menopausal Daughters and Mothers

Radial (MRad) aBMD did not show relational significance within this pair [46], but hip (FN) aBMD did [23]. 

##### Pre-Menopausal Daughters and Post-Menopausal Mothers

There was a 1:1 ([32]:[48]) ratio for WB results. Forearm BMC measured by Hansen, Hassager [22], was significantly related in both of their populations. Adjacently, radial results were significant for all Nagy, Sornay-Rendu [38] sites in their 2013 paper (DXA: UDRad, MDRad and MRad, and HR-pQCT UDRad), but two other groups’ studies were not significant at the mid-site [32,46]. The lumbar spine results ratio was 7:3 (LS [36,38,43,44], and L2–4 [26] and both Hansen, Hassager [22] populations: LS [32] and L2–4 [34,40]). 

The hip category included fifteen significant results (TH [26,36,38], FN [22,26,34,36,38,44], Troch [22,36,43], iTroch [22] and Ward [34]) and only five without significance (TH [43], FN [32,43], Troch [34] and Ward [43]). Seeman, Hopper [44] did not find significant similarity at the femur. Conversely, at the distal tibia, Nagy, Sornay-Rendu [38] did report a significant result. Areal BMD (measured by SXA and SPA) at the heel did show a significant resemblance [26,32], but BUA (from QUS) was 1:1 ([27]:[26]).

##### Mixed-Menopausal Daughters and Post-Menopausal Mothers

The only study to investigate these pairs and report correlation or regression results was conducted by Danielson and Cauley [26]. They saw a significant relationship at the lumbar spine (L2–4), but not at the hip (FN and TH). They also did not see one at the heel through either SXA (aBMD) or QUS (BUA). 

##### Post-Menopausal Daughters and Mothers

Conversely, when Danielson and Cauley [26] only looked at the pairs with post-menopausal daughters, the relationship was no longer significant at the lumbar spine (L2–4) but it was at the heel (both aBMD and BUA) and the TH. The FN remained non-significant.

##### Adolescent/Young Adult-Aged Daughters and Post-Menopausal Grandmothers

Ohta and Kuroda [40] reported no significant results for L2–4 between adolescent/young adult granddaughters and their post-menopausal grandmothers.

#### 3.5.3. Familial Bone Heritability

**Figure 5:**
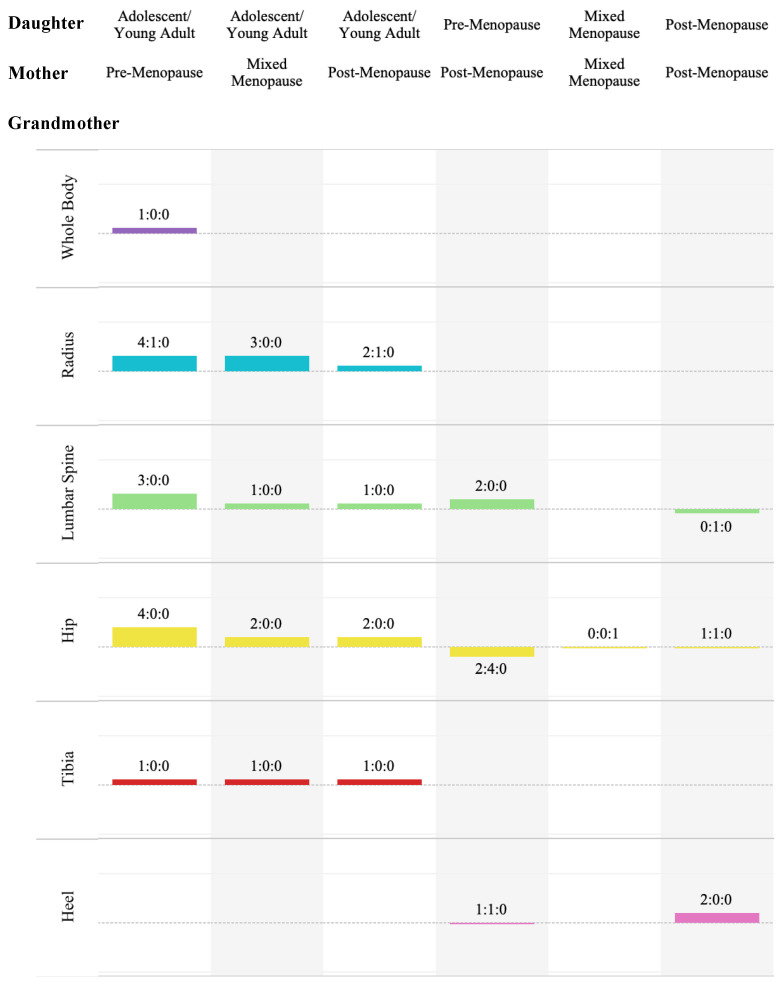
Sum and ratio (sig:non:unknown) of significant (+1) and non-significant (−1) heritability results by pairing and ROI category.

The ratios of reportedly significant and non-significant heritability can be seen in Figure 5 and Table S12. As heritability is derived from regression analyses, it is important to note that non-significant regression or correlation results were likely not analyzed for heritability. Thus, heritability results should be considered more as a sub-analysis of correlation and regression than a distinct test. There were no applicable results for the femur or forearm, nor were there any applicable results for pre-menopausal daughters and mothers, pre-menopausal daughters and mixed-menopausal mothers, mixed-menopausal daughters and post-menopausal mothers, adolescent daughters and post-menopausal grandmothers, or triads. 

##### Adolescent/Young Adult-Aged Daughters and Pre-Menopausal Mothers

Heritability was statistically significant for the whole body [37], lumbar spine (LS [37,41] and L2–4 [29]), hip (TH [37,41] and FN [37,41]) and tibia [41] ROIs within this pairing. The radius mostly displayed significant heritability (UDRad [41], MDRad [47] and MRad [41,47]), though not entirely (UDRad [41]). 

##### Adolescent/Young Adult-Aged Daughters and Mixed-Menopausal Mothers

All results within this pairing came from Pepe in 2017 [41] and were significant (radius—UDRad aBMD and vBMD, MRad aBMD, LS aBMD; hip—TH and FN aBMD; and Distal Tibia vBMD). 

##### Mixed-Menopausal Daughters and Mothers

There was only one ROI result for this pairing, by Blain and Vuillemin [23]; it was at the FN, and it was unclear.

##### Adolescent/Young Adult-Aged Daughters and Post-Menopausal Mothers

Pepe and Biver [41] were the sole group to investigate heritability in this pairing. It was significant for the DXA-derived aBMD ROIs at the hip (TH and FN), LS and radius (UDRad and MRad). The HR-pQCT-derived total vBMD of the distal tibia was significantly heritable, but the ultra-distal radius total vBMD was not.

##### Pre-Menopausal Daughters and Post-Menopausal Mothers

Significant heritability was reported for the lumbar spine (LS [26,43]). The hip’s heritability results had a ratio of 2:4 (TH and FN [26]: TH, FN, Troch and Ward [43]). There was one significant (aBMD [26]) and one not significant (BUA [26]) result for the heel.

##### Post-Menopausal Daughters and Mothers

Danielson and Cauley [26] reported significant heritability results for two heel measures (BUA and aBMD) and one hip ROI (TH), as well as non-significant results for L2–4 and another hip ROI (FN). 

## 4. Discussion

This is the first scoping review, to the best of our knowledge, to examine and compile the literature regarding bone health parameters in sexually mature daughters, mothers and grandmothers. This review aimed to investigate how (grandmother–)mother–daughter and mother–daughter bone health studies were conducted and how the literature has reported the comparisons and relationships in bone health parameters between maternal triads and pairs. This approach was challenging due, in part, to the varying ages and imaging technologies used across the studies. However, we bring forward several commonalities, challenges and trends in the methodology and results that have emerged throughout this process. 

The most used scanning modality within the included papers, singularly or in conjuncture with others, was DXA, which is not surprising as it has been considered the “gold standard” for assessing the mass and mineral aspects of bone quality. It is highly validated [9], with abundant and convenient reference ranges [58]. The least common was SXA, which was only used once [26]. DXA is more precise than DPA [59] and images a greater range of ROIs than SXA or SPA [9]. Although HRpQCT and pQCT are also limited in their scanned ROIs, they provide greater insight into the microarchitecture of the bone than DXA or the other modalities [60]. The vBMD, assessed through pQCT and HR-pQCT, and the aBMD, from DXA, have also been shown to be correlated in healthy [61,62,63] and diseased populations [64]. 

Seventeen studies reported results for at least one of the hip ROIs and, therefore, this was the most imaged body segment, regardless of scanning modalities. This makes sense for a few reasons. The first reason is that all five of the hip ROIs can be captured within one hip scan through modalities such as DXA. This is cost- and time-effective if you want to capture multiple regions. Additionally, the International Society for Clinical Densitometry (ISCD) states that the lowest T-score from TH, FN, Troch or LS (preferably L1–4 if possible), should be used in the diagnosis of osteoporosis [65]. This is similar to the World Health Organization’s diagnostic recommendations [66], which demonstrates the alignment between the interests of the clinical and research communities. Unlike the hip, the lumbar spine category only contains two ROIs, (L1–4 and L2–4) but is the second most reported region (15 studies). It is also a region commonly highlighted in guidelines (as seen above with the ISCD). 

Another reason the hip was so popular was due to the hip’s importance regarding fractures. An individual’s 10-year fracture risk is commonly assessed using their FRAX score, which has been tailored to various countries including Canada [67]. FRAX uses an individual’s FN aBMD T-score to increase the accuracy of fracture prediction [67]. Not only is hip bone health important for the prediction of fractures but it is also the target for preventing fractures, as both hip and spinal fractures are the most common osteoporotic fractures. Therefore, these sites being the most reported across the papers of this scoping review indicate a possible targeting of these methodologies to increase their clinical applicability. 

Within international (e.g., ICSD [65]) and national (e.g., Canadian 2023 clinical practice guidelines [67]) osteoporosis-related recommendations and guidelines, menopausal status is frequently considered alongside bone health quantification. This reflects the pivotal role menopause has on female bone health. The pairing patterns observed within this review similarly reflect a concerted effort to investigate this developmental milestone. The most common mother–daughter pairing was pre-menopausal daughters and post-menopausal mothers (n = 18). This family pairing design, which leverages the high heritability of bone variance [11,15], allows researchers to investigate menopause without having to follow women longitudinally. Looking at the bone health of daughters provides an estimate of their mothers’ historic bone health parameters, and the mothers’ measures provide insight into the daughters’ potential trajectory. 

The second most-seen pair was late adolescent/young adult-aged daughters and pre-menopausal mothers (n = 12). This highlights another developmental period which is critical for bone health, the puberty-to-adulthood transition. Women appear to reach peak bone mass between the ages of 18 yrs [12] and 40 yrs [13], which varies depending on the bone or skeletal region. Roughly one-third of an individual’s peak bone mass is accrued in just the four years surrounding puberty, and accrual is over 95% complete in females by age 20 [12]. The relationship between this dynamic period of growth and various lifestyle factors is important because PBM is a predictor of future low bone mass or fracture [9]. An increase in accrual can theoretically delay the age at which osteoporosis is developed [68]. Late adolescent and young adult daughters have built much of their total adult bone mass. They and their pre-menopausal mothers are an ideal representation of the period in adulthood where bone is the most stable but near periods of large-scale change.

In addition, different bone ROIs have been shown to accrue and lose bone at different times. Regarding PBM, in late adolescence/young adulthood, the hip region(s) plateau before the lumbar spine [12]. Conversely, during late mixed-menopause, and into post-menopause, Finkelstein and Brockwell [69] found a greater rate of bone loss at the lumbar spine than at the hip. This furthers the importance of these maturational periods and bone ROIs, and it supports the popularity of investigating them.

There were several different ways in which the various studies handled a mother paired with multiple daughters. However, some, like Nagy and Sornay-Rendu [38] and Nagy and Chapurlat [39], did not account for this throughout all of their analyses. Not accounting for the correlations between siblings (by counting each sibling–mother pair as independent from each other) overestimates significance [57]. Rosner and Donner [57] compared four (three known and one proposed) methods for statistically handling siblingships within one parent, and they showed that, among the three known methods, using the arithmetic mean of the siblings is the least likely to inflate their significance. Their proposed method, used by Sowers and Burns [46], proved no worse than the sibling mean method, but allows for superior power. Though not used by any included study, hierarchical modelling is another possible method that can be used. It accounts for the clustering of siblings within their “parent”, therefore taking into account their inherent environmental and genetic commonalities [70].

Heritability estimates among the reported studies were largely significant; this is likely because heritability is a parameter derived from the regression analysis. In their methods, Runyan and colleagues [43] indicate that they only reported the regression analyses in which the mother’s BMD was a significant factor. It is possible that other authors similarly only reported the regression analyses, and subsequent heritability statistics, for significant tests. Which may account for the high proportion of these significant results.

Heritability is a concept with three distinct definitions and interpretations, and, as such, it can be confused and misused readily [71]. ‘Biometric heritability’ (h^2^) is the least complex of the three [71,72] and was the most reported among these studies [23,26,29,43]. This heritability indicates the degree of parent–offspring resemblance in a measured characteristic with no explanation for the mechanism of this resemblance; however, the other two methodologies attempt to account for the genetic and/or environmental aspects of the heritability [71]. Not used in the included studies is ‘narrow sense heritability’ (h^2^_N_), which is the most complex, most theoretical, and least applicable of the three [71]. ‘Broad sense heritability’ (h^2^_L_), used in three studies [37,41,47], accounts for variance in a character due to the additive genetics between family members. It is complicated by assumptions of similarity in environmental factors and their effect [71,72]. Falconer [72] indicates that maternal–offspring pairs may show over-estimated h^2^_L_, as gestation increases exposure to similar environmental factors, which may have impacted the studies [37,41,47] that utilized this methodology. However, regardless of which heritability methodology was used, significance was often seen.

Correlation and regression statistics demonstrated a significant relationship between the daughters and mothers’ bone quality among all included studies, ROIs and pairings. The significance seen across age/reproductive status groups and across pairings supports the highly heritable nature of bone health parameters [11,15]. The familial resemblance that was determined by early studies and elaborated upon and clarified through the subsequent years supports the use of the parent–offspring model to investigate bone health pseudo-longitudinally. One included example of this was from Wang and Xu [49]. They investigated whether pubertal bone accrual (in daughters) occurred in the same sequence as bone loss (in grandmothers). Differences in bone quality were most often reported as being significant within the included studies across scanning ROIs and daughter–mother pairings. So, even though there is familial resemblance, as displayed through correlation, regression and heritability analyses, there are still differences between the mother–daughter pairs’ bones. This is possibly due to the 20–40% of bone variance than is not heritable [11,15]. 

### Strength and Limitations

One strength of this review is the collection and simplified synthesis of the results of these studies. The difficulty of compiling results across imaging modalities and regions of interest was undertaken by these authors, thus decreasing the time and effort required to understand these trends in the future. 

The simplicity of synthesizing the results based solely on whether they were significant or not allows the results to be viewed as an overview or synopsis of trends. However, no assessment of result quality was undertaken before this synthesis, which limits the strength and depth of the possible conclusions that can be drawn. It must be acknowledged that the information within this review is limited to the reporting of the original studies’ authors. An example of this was the exclusion of several studies due to the lack of information regarding participants’ menopausal status. 

This review highlights important methodological commonalities and challenges related to compiling imaging studies that represent mother–daughter bone parameters, the compilation of which can be used to increase the efficiency and efficacy of conducting future studies. The gaps in the literature shown in this review, such as the lack of microarchitectural comparisons across most pairs, indicate areas for future study. A final strength of the review is that it highlights the importance and complexity of investigating female bones across their entire lifespan. If not the entire lifespan, then the obvious importance of menopause and peak bone mass as developmental milestones relating to bone become clear. 

## 5. Conclusions

The imaging characteristics of the included studies match reasonably well with current clinical recommendations and guidelines. Additionally, these bone quantification methodological features, as well as those of the included participants, appear to be tailored towards clinical applicability. Not only does this increase the real-world applicability of these studies, but it also engenders comparisons and a consolidation of study results. 

The results from these studies, and the trends seen through the ratios, further the importance of menopause within female skeletal aging and may be a potential indicator, or additional puzzle piece, that can be used when assessing the bone-related risks that young females face. This information could also provide some clarity for an individual’s bone health trajectory. Further studies should consider following these pairings to evaluate whether these trends track over time. Ideally, this could be carried out across the PBM or menopausal time points. Including pQCT and HR-pQCT modalities will further enable the elucidation of these aging and reproductive trends and clarify them within the bones’ microarchitecture. 

## Figures and Tables

**Table 1:** Inclusion and exclusion criteria by PCC category, sub-category, and criteria.

PCC Category	Sub-Category	Criteria
Population	Female	Female sex
	Pairs	Biologically related parent and offspring.
	Reproductive	All members of pairs/triads had to have passed menarche. ^a^ Menopause had to be determined and accounted for in the study design (through grouping) or in the analysis (by adjusting for it). ^a^
	Medical	Had to have screened and accounted for medical conditions or treatments that could affect bone. ^b^
Concept	Familial Resemblance	Relationship between mother and daughters’ bone health had to be analyzed statistically (e.g., correlation, regression, *t*-tests). ^a^
	Bone Health	Bone health/quality had to be quantified through an imaging modality. ^a^
Context	Design, Setting, Publication Type	No limits were placed upon study setting. Other than case studies/reports and non-original research papers (i.e., reviews), no study designs were excluded. Publications such as opinion papers, editorials and obituaries were excluded, but not grey literature.
	Language, Country, Year	No exclusions were made for publishing language, country or year.

^a^ Protocol clarification; ^b^ Screening was required but did not need to exclude participants.

**Table 4:** Study characteristics by region, year, imaging modality, ROI category and study counts.

Study Characteristics (N = 29)	Count (Studies)	
Population Continent		
North America	9	[26,32,34,36,42,43,46,47,48]
Europe	13	[22,23,25,27,28,29,31,38,39,41,49,50,51]
Oceania	3	[30,35,44]
Asia	4	[33,37,40,45]
Publication Year		
1985–1989	3	[44,46,47]
1990–1994	4	[22,32,34,36]
1995–1999	6	[26,29,30,31,35,48];
2000–2004	3	[27,42,43];
2005–2009	3	[23,25,28];
2010–2014	5	[37,38,40,49,51];
2015–2019	4	[39,41,45,50];
2020	1	[33]
Imaging Modality		
DXA	18	[22,23,25,26,28,30,31,32,33,36,37,38,39,41,43,45,48,50]
DPA	5	[29,32,34,42,44]
SPA	5	[22,32,35,46,47]
HR-pQCT	3	[38,39,41]
pQCT	2	[49,51]
QUS	2	[26,27]
SXA	1	[26]
ROI Category: # of ROIs		
Whole Body (WB): 1 ROI	7	[25,26,31,32,33,37,48]
Radius (Rad): 3 ROIs	8	[28,32,38,39,41,46,47,49]
Forearm: 1 ROI	2	[22,35]
Lumbar Spine (LS): 2 ROIs	15	[22,26,28,29,30,32,34,36,37,38,39,40,41,42,43]
Hip: 5 ROIs	17	[22,23,26,28,30,32,33,34,36,37,38,39,41,43,44,45,50]
Femur (Fem): 1 ROI	3	[28,30,44]
Tibia (Tib): 2 ROIs	5	[38,39,41,49,51]
Heel/Calcaneus: 1 ROI	3	[26,27,32]

ROI: region of interest. DXA: dual-energy X-ray absorptiometry. DPA: dual photon absorptiometry. SPA: single photon absorptiometry. pQCT: peripheral quantitative computed tomography. HR-pQCT: high-resolution pQCT. QUS: quantitative ultrasound. SXA: single X-ray absorptiometry.

**Table 5:** Bone imaging characteristics (modality, scanned regions of interest and ROI category) of studies.

Study	Scan Modalities									Radius (Rad)			Lumbar Spine (LS)		Hip						Tibia (Tib)		
Author, Year	DXA	DPA	SPA	HR-pQCT	pQCT	QUS	SXA	Whole Body	Forearm	Ultra-Distal (UDRad)	Mid-Distal (MDRad)	Mid (MRad)	LS (L1–4)	L2–4	Total Hip (TH)	Femoral Neck (FN)	Trochanter (Troch)	Inter-Trochanter (iTroch)	Ward’s Area (Wards)	Femur (Fem): Mid-Shaft/Diaphysis	Proximal (Prox)	Distal (Dist)	Heel
Blain et al., 2006 [23]	X															X DXA: aBMD							
Cheng et al., 2009 [25]	X							X DXA: BM (kg)															
Danielson et al., 1999 [26]	X					X	X	X DXA: aBMD					X DXA: aBMD		X DXA: aBMD	X DXA: aBMD							X SXA: aBMD QUS: BUA
Drozdzowska and Pluskiewicz, 2001 [27]						X																	X QUS: BUA
Ferrari et al., 2006 [28]	X									X DXA: BMC		X DXA: BMC		X DXA: BMC		X DXA: BMC	X DXA: BMC			X DXA: BMC			
Francois et al., 1999 [29]		X												X DPA: aBMD									
Hansen et al., 1992 [22]	X		X						X ^c^ SPA: BMC					X ^d^ DXA: aBMD		X ^e^ DXA: aBMD	X ^e^ DXA: aBMD	X ^e^ DXA: aBMD					
Henderson et al., 1995 [30]	X												X DXA: aBMD		X DXA: aBMD	X DXA: aBMD	X DXA: aBMD	X DXA: aBMD		X DXA: aBMD			
Jouanny et al., 1995 [31]	X							X DXA: aBMD															
Krall and Dawson-Hughes, 1993 [32] ^a^	X	X	X					X DXA: aBMD				X SPA: aBMD		X DXA: aBMD		X DXA: aBMD							X SPA: aBMD
Lee et al., 2020 [33]								X DXA: aBMD					X DXA: aBMD		X DXA: aBMD	X DXA: aBMD							
Lutz and Tesar, 1990 [34]		X											X DPA: aBMD	X DPA: aBMD		X DPA: aBMD	X DPA: aBMD		X DPA: aBMD				
Margarey et al., 1999 [35]			X						X SPA: vBMD														
McKay et al., 1994 [36]	X												X DXA: aBMD		X DXA: aBMD	X DXA: aBMD	X DXA: aBMD						
Nabulsi et al., 2013 [37]	X							X DXA: aBMD and Z-score					X DXA: aBMD and Z-score		X DXA: aBMD and Z-score	X DXA: aBMD and Z-score							
Nagy et al., 2013 [38]	X			X						X DXA: aBMD HR-pQCT: Tt.vBMD	X DXA: aBMD	X DXA: aBMD	X DXA: aBMD		X DXA: aBMD	X DXA: aBMD						X DXA: aBMD HR-pQCT: Tt.vBMD	
Nagy et al., 2015 [39]	X			X						X DXA: aBMD HR-pQCT: Tt.vBMD			X ^b^ DXA		X DXA: aBMD	X DXA: aBMD						X DXA: aBMD HR-pQCT: Tt.vBMD	
Ohta et al., 2010 [40]	X													X DXA: aBMD								X HR-pQCT: Tt.vBMD	
Pepe et al., 2017 [41]	X			X						X DXA: aBMD HR-pQCT: Tt.vBMD		X DXA: aBMD		X DXA: aBMD	X DXA: aBMD	X DXA: aBMD							
Picard et al., 2001 [42]		X												X DXA: aBMD									
Runyan et al., 2003 [43]	X												X DXA: aBMD		X DXA: aBMD	X DXA: aBMD	X DXA: aBMD		X DXA: aBMD				
Seeman et al., 1989 [44]		X												X DPA: BM(g)		X DPA: aBMD				X DPA: BMC (g/cm)			
Shetty et al., 2016 [45]	X												X DXA: aBMD			X DXA: aBMD							
Sowers et al., 1986 [46]			X									X SPA: aBMD											
Tylvasky et al., 1989 [47]			X								X SPA: aBMD	X SPA: aBMD											
Ulrich et al., 1996 [48]	X							X DXA: aBMD															
Wang et al., 2011 [49]					X						X pQCT: Tt.vBMD										X pQCT: Tt.vBMD		
Wang et al., 2015 [50]	X														X DXA: aBMD	X DXA: aBMD							
Xu et al., 2010 [51]					X																X pQCT: Ct.vBMD		

Some studies may include further measures (e.g., bone mineral content, bone mass or bone microarchitecture variables). This table only includes those that went towards our result ratios. ^a^: study primarily uses DXA, but some participants were from a previous study using DPA. Their baseline data come from the previous study. ^b^: methods include this ROI, but were not included in results. Hansen 1992 [22]: ^c^—All mothers and daughters in both populations, as well as population 1 mothers’ historical data. ^d^—all mothers and daughters, in both populations, but not population 1 mothers’ historical data. ^e^— all daughters, in populations 1 and 2 but only population 1 mothers. ROI: region of interest. DXA: dual-energy X-ray absorptiometry. DPA: dual photon absorptiometry. SPA: single photon absorptiometry. pQCT: peripheral quantitative computed tomography. HR-pQCT: high-resolution pQCT. QUS: quantitative ultrasound. SXA: single X-ray absorptiometry. aBMD: areal bone mineral density. vBMD: volumetric bone mineral density. BMC: bone mineral content. BM: bone mass. Tt.vBMD: total (cortical and trabecular) volumetric bone mineral density. Ct.vBMD: cortical volumetric bone mineral density. BUA: broadband ultrasound attenuation.

## Data Availability

The search is available in the Supplementary Materials.

## Supplementary Materials

The following supporting information can be downloaded at https://www.mdpi.com/article/10.3390/life14070819/s1, Table S1: Preferred Reporting Items for Systematic reviews and Meta-Analyses extension for Scoping Reviews (PRISMA-ScR) checklist; Tables S2–S7: Search strategies for Medline, Embase, CENTRAL, CINAHL Plus with Full Text, SportDiscus and SCOPUS; Table S8: Grey literature conference search; Table S9: Categorization and definition of regions of interest (ROIs); Tables S10–S12: Ratios of significant/non-significant (/unknown significance) differences, correlations/regressions and heritability. Figure S1: Radial ROI categorizations. Reference [73] is cited in the Supplementary Materials.
