# Supplementary material for: Familial Resemblance of Bone Health in Maternal Lineage Pairs and Triads: A Scoping Review
Source: Life (Basel). 2024 Jun 27;14(7):819. doi: 10.3390/life14070819 (PMC11277880; doi:10.3390/life14070819)
Supplement: Supplementary file 1 [file life-14-00819-s001.zip › life-3044270-supplementary.pdf]

Supplementary 1: PRISMA Checklist

Table S1: Preferred Reporting Items for Systematic reviews and Meta-Analyses extension for Scoping Reviews (PRISMA-ScR) Checklist

SECTION	ITEM	PRISMA-ScR CHECKLIST ITEM	REPORTED ON PAGE #
**TITLE**			
Title	1	Identify the report as a scoping review.	1 (of article)
**ABSTRACT**			
Structured summary	2	Provide a structured summary that includes (as applicable): background, objectives, eligibility criteria, sources of evidence, charting methods, results, and conclusions that relate to the review questions and objectives.	1
**INTRODUCTION**			
Rationale	3	Describe the rationale for the review in the context of what is already known. Explain why the review questions/objectives lend themselves to a scoping review approach.	1-2
Objectives	4	Provide an explicit statement of the questions and objectives being addressed with reference to their key elements (e.g., population or participants, concepts, and context) or other relevant key elements used to conceptualize the review questions and/or objectives.	2
**METHODS**			
Protocol and registration	5	Indicate whether a review protocol exists; state if and where it can be accessed (e.g., a Web address); and if available, provide registration information, including the registration number.	3
Eligibility criteria	6	Specify characteristics of the sources of evidence used as eligibility criteria (e.g., years considered, language, and publication status), and provide a rationale.	2-3
Information sources*	7	Describe all information sources in the search (e.g., databases with dates of coverage and contact with authors to identify additional sources), as well as the date the most recent search was executed.	3&7 (& Supplemental 2)
Search	8	Present the full electronic search strategy for at least 1 database, including any limits used, such that it could be repeated.	Supplemental 2

SECTION	ITEM	PRISMA-ScR CHECKLIST ITEM	REPORTED ON PAGE #
Selection of sources of evidence†	9	State the process for selecting sources of evidence (i.e., screening and eligibility) included in the scoping review.	3 & 5
Data charting process‡	10	Describe the methods of charting data from the included sources of evidence (e.g., calibrated forms or forms that have been tested by the team before their use, and whether data charting was done independently or in duplicate) and any processes for obtaining and confirming data from investigators.	4-5
Data items	11	List and define all variables for which data were sought and any assumptions and simplifications made.	4-5
Critical appraisal of individual sources of evidence§	12	If done, provide a rationale for conducting a critical appraisal of included sources of evidence; describe the methods used and how this information was used in any data synthesis (if appropriate).	NA
Synthesis of results	13	Describe the methods of handling and summarizing the data that were charted.	4-5
**RESULTS**			
Selection of sources of evidence	14	Give numbers of sources of evidence screened, assessed for eligibility, and included in the review, with reasons for exclusions at each stage, ideally using a flow diagram.	5
Characteristics of sources of evidence	15	For each source of evidence, present characteristics for which data were charted and provide the citations.	6-14
Critical appraisal within sources of evidence	16	If done, present data on critical appraisal of included sources of evidence (see item 12).	NA
Results of individual sources of evidence	17	For each included source of evidence, present the relevant data that were charted that relate to the review questions and objectives.	6-19
Synthesis of results	18	Summarize and/or present the charting results as they relate to the review questions and objectives.	6-19
**DISCUSSION**			
Summary of evidence	19	Summarize the main results (including an overview of concepts, themes, and types of evidence available), link to the review	19-21

SECTION	ITEM	PRISMA-ScR CHECKLIST ITEM	REPORTED ON PAGE #
		questions and objectives, and consider the relevance to key groups.	
Limitations	20	Discuss the limitations of the scoping review process.	21
Conclusions	21	Provide a general interpretation of the results with respect to the review questions and objectives, as well as potential implications and/or next steps.	22
**FUNDING**			
Funding	22	Describe sources of funding for the included sources of evidence, as well as sources of funding for the scoping review. Describe the role of the funders of the scoping review.	NA

JBI = Joanna Briggs Institute; PRISMA-ScR = Preferred Reporting Items for Systematic reviews and Meta-Analyses extension for Scoping Reviews.

* Where *sources of evidence* (see second footnote) are compiled from, such as bibliographic databases, social media platforms, and Web sites.

† A more inclusive/heterogeneous term used to account for the different types of evidence or data sources (e.g., quantitative and/or qualitative research, expert opinion, and policy documents) that may be eligible in a scoping review as opposed to only studies. This is not to be confused with *information sources* (see first footnote).

‡ The frameworks by Arksey and O'Malley (6) and Levac and colleagues (7) and the JBI guidance (4, 5) refer to the process of data extraction in a scoping review as data charting.

§ The process of systematically examining research evidence to assess its validity, results, and relevance before using it to inform a decision. This term is used for items 12 and 19 instead of "risk of bias" (which is more applicable to systematic reviews of interventions) to include and acknowledge the various sources of evidence that may be used in a scoping review (e.g., quantitative and/or qualitative research, expert opinion, and policy document).

From:[1] Tricco AC, Lillie E, Zarin W, O'Brien KK, Colquhoun H, Levac D, et al. PRISMA Extension for Scoping Reviews (PRISMAScR): Checklist and Explanation. Ann Intern Med. 2018;169:467–473. doi: 10.7326/M18-0850.

Supplementary 2: Search Strategy

Medline
May 6, 2021

Database(s): **Ovid MEDLINE(R) and Epub Ahead of Print, In-Process, In-Data-Review & Other Non-Indexed Citations and Daily** 1946 to May 05, 2021.
Table S2: Medline Search Strategy:

#	Searches	Results
1	Mothers/	46065
2	Nuclear Family/	5646
3	Family/	78103
4	Family Health/	23641
5	(familial adj3 (correlation* or resemblance* or pattern* or characteristic* or similarit* or component* or lineage)).tw,kf.	3024
6	(mother* adj6 daughter*).tw,kf.	5119
7	(mother* adj6 (grand-mother* or grandmother*)).tw,kf.	1070
8	(daughter* adj6 (grand-mother* or grandmother*)).tw,kf.	101
9	((grand-daughter* or granddaughter* or grandaughter*) adj6 (grand-mother* or grandmother*)).tw,kf.	44
10	((two or three or "2" or "3" or triad*) adj2 generation*).tw,kf.	20209
11	((family or familial) adj6 (pair* or triad*)).tw,kf.	2229
12	((mother* or daughter* or grand-mother* or grandmother* or grand-daughter* or granddaughter* or grandaughter*) adj3 (pair* or triad*)).tw,kf.	7016
13	(multi-generation* or multigeneration* or inter-generation* or intergeneration* or bi-generation* or bigeneration* or tri-generation* or trigeneration*).tw,kf.	10523
14	or/1-12	181468
15	Bone Density/	54960
16	osteoporosis/ or osteoporosis, postmenopausal/	57394
17	exp Fractures, Bone/	189483
18	(bone adj3 (densit* or health or mass or mineral* or content* or fracture* or characteristic* or qualit*)).tw,kf.	113834
19	bmd.tw,kf.	30787
20	(osteoporosis or osteopenia).tw,kf.	77868

21	(fracture* adj2 (risk* or history or bone*)).tw,kf.	32791
22	(dual-energy adj2 absorptiometry).tw,kf.	424
23	(DXA or DEXA or FRAX).tw,kf.	17281
24	or/15-23	339696
25	14 and 24	696

Embase
May 9, 2021

Database(s): **Embase** 1974 to 2021 May 07
Table S3: Embase Search Strategy:

#	Searches	Results
1	mother/ or daughter/	93662
2	family/ or family health/ or grandchild/ or grandparent/ or nuclear family/	110258
3	(familial adj3 (correlation* or resemblance* or pattern* or characteristic* or similarit* or component* or lineage)).tw,kw.	3747
4	(mother* adj6 daughter*).tw,kw.	6352
5	(mother* adj6 (grand-mother* or grandmother*)).tw,kw.	1613
6	(daughter* adj6 (grand-mother* or grandmother*)).tw,kw.	138
7	((grand-daughter* or granddaughter* or grandaughter*) adj6 (grand-mother* or grandmother*)).tw,kw.	57
8	((two or three or "2" or "3" or triad*) adj2 generation*).tw,kw.	23775
9	((family or familial) adj6 (pair* or triad*)).tw,kw.	2690
10	((mother* or daughter* or grand-mother* or grandmother* or grand-daughter* or granddaughter* or grandaughter*) adj3 (pair* or triad*)).tw,kw.	8690
11	(multi-generation* or multigeneration* or inter-generation* or intergeneration* or bi-generation* or bigeneration* or tri-generation* or trigeneration*).tw,kw.	12168
12	or/1-11	248416
13	bone density/	99912
14	osteoporosis/ or postmenopause osteoporosis/	133013
15	exp fracture/	307539
16	(bone adj3 (densit* or health or mass or mineral* or content* or fracture* or characteristic* or qualit*)).tw,kw.	163378

17	bmd.tw,kw.	52964
18	(osteoporosis or osteopenia).tw,kw.	125960
19	(fracture* adj2 (risk* or history or bone*)).tw,kw.	49812
20	(dual-energy adj2 absorptiometry).tw,kw.	749
21	(DXA or DEXA or FRAX).tw,kw.	37310
22	or/13-21	519344
23	12 and 22	1811

CENTRAL
May 9, 2021

Database(s): **EBM Reviews - Cochrane Central Register of Controlled Trials** April 2021
Table S4: Central Search Strategy:

#	Searches	Results
1	Mothers/	1890
2	Nuclear Family/	83
3	Family/	1516
4	Family Health/	434
5	(familial adj3 (correlation* or resemblance* or pattern* or characteristic* or similarit* or component* or lineage)).tw,kw.	53
6	(mother* adj6 daughter*).tw,kw.	186
7	(mother* adj6 (grand-mother* or grandmother*)).tw,kw.	63
8	(daughter* adj6 (grand-mother* or grandmother*)).tw,kw.	4
9	((grand-daughter* or granddaughter* or grandaughter*) adj6 (grand-mother* or grandmother*)).tw,kw.	0
10	((two or three or "2" or "3" or triad*) adj2 generation*).tw,kw.	593
11	((family or familial) adj6 (pair* or triad*)).tw,kw.	108
12	((mother* or daughter* or grand-mother* or grandmother* or grand-daughter* or granddaughter* or grandaughter*) adj3 (pair* or triad*)).tw,kw.	827
13	(multi-generation* or multigeneration* or inter-generation* or intergeneration* or bi-generation* or bigeneration* or tri-generation* or trigeneration*).tw,kw.	234
14	or/1-12	5433
15	Bone Density/	4682

16	osteoporosis/ or osteoporosis, postmenopausal/	4167
17	exp Fractures, Bone/	6155
18	(bone adj3 (densit* or health or mass or mineral* or content* or fracture* or characteristic* or qualit*)).tw,kw.	16984
19	bmd.tw,kw.	6420
20	(osteoporosis or osteopenia).tw,kw.	10358
21	(fracture* adj2 (risk* or history or bone*)).tw,kw.	5078
22	(dual-energy adj2 absorptiometry).tw,kw.	100
23	(DXA or DEXA or FRAX).tw,kw.	4782
24	or/15-23	29353
25	14 and 24	34

CINAHL Plus with Full Text
May 9, 2021

Table S5: CINAHL Plus with Full Text Search Strategy: *Search modes – Find all my search terms*

#	Query	Results
S1	(MH "Mothers") or (MH "Daughters") or (MH "Grandparents")	34,504
S2	(MH "Family") OR (MH "Nuclear Family")	44,428
S3	TI ((familial N3 (correlation* or resemblance* or pattern* or characteristic* or similarit* or component* or lineage))) OR AB ((familial N3 (correlation* or resemblance* or pattern* or characteristic* or similarit* or component* or lineage)))	625
S4	TI (mother* N6 daughter*) OR AB (mother* N6 daughter*)	1,774
S5	TI ((mother* N6 (grand-mother* or grandmother*))) OR AB ((mother* N6 (grand-mother* or grandmother*)))	438
S6	TI ((daughter* N6 (grand-mother* or grandmother*))) OR AB ((daughter* N6 (grand-mother* or grandmother*)))	51
S7	TI ((((grand-daughter* or granddaughter* or grandaughter*) N6 (grand-mother* or grandmother*))) OR AB (((grand-daughter* or granddaughter* or grandaughter*) N6 (grand-mother* or grandmother*)))	23
S8	TI (((two or three or "2" or "3" or triad*) N2 generation*)) OR AB (((two or three or "2" or "3" or triad*) N2 generation*))	2,250
S9	TI (((family or familial) N6 (pair* or triad*))) OR AB (((family or familial) N6 (pair* or triad*)))	664

S10	TI (((mother* or daughter* or grand-mother* or grandmother* or grand-daughter* or granddaughter* or grandaughter*) N3 (pair* or triad*))) OR AB (((mother* or daughter* or grand-mother* or grandmother* or grand-daughter* or granddaughter* or grandaughter*) N3 (pair* or triad*)))	2,317
S11	TI ((multi-generation* or multigeneration* or inter-generation* or intergeneration* or bi-generation* or bigeneration* or tri-generation* or trigeneration*)) OR AB ((multi-generation* or multigeneration* or inter-generation* or intergeneration* or bi-generation* or bigeneration* or tri-generation* or trigeneration*))	5,176
S12	S1 OR S2 OR S3 OR S4 OR S5 OR S6 OR S7 OR S8 OR S9 OR S10 OR S11	87,201
S13	(MH "Bone Density")	18,424
S14	(MH "Osteoporosis")	22,841
S15	(MH "Fractures+")	60,689
S16	TI ((bone N3 (densit* or health or mass or mineral* or content* or fracture* or characteristic* or qualit*))) OR AB ((bone N3 (densit* or health or mass or mineral* or content* or fracture* or characteristic* or qualit*)))	27,581
S17	TI bmd OR AB bmd	6,684
S18	TI ((osteoporosis or osteopenia)) OR AB ((osteoporosis or osteopenia))	19,644
S19	TI ((fracture* N2 (risk* or history or bone*))) OR AB ((fracture* N2 (risk* or history or bone*)))	11,913
S20	TI (dual-energy N2 absorptiometry) OR AB (dual-energy N2 absorptiometry)	6,418
S21	TI ((DXA or DEXA or FRAX)) OR AB ((DXA or DEXA or FRAX))	4,383
S22	S13 OR S14 OR S15 OR S16 OR S17 OR S18 OR S19 OR S20 OR S21	103,320
S23	S12 AND S22	260

SportDiscus
May 9, 2021

Table S6: SportDiscus Search Strategy: *Search modes – Find all my search terms*

#	Query	Results
S1	TI ((familial N3 (correlation* or resemblance* or pattern* or characteristic* or similarit* or component* or lineage))) OR AB ((familial N3 (correlation* or resemblance* or pattern* or characteristic* or similarit* or component* or lineage))) OR KW ((familial N3 (correlation* or resemblance* or pattern* or characteristic* or similarit* or component* or lineage)))	116
S2	TI (mother* N6 daughter*) OR AB (mother* N6 daughter*) OR KW (mother* N6 daughter*)	346

S3	TI ((mother* N6 (grand-mother* or grandmother*))) OR AB ((mother* N6 (grand-mother* or grandmother*))) OR KW ((mother* N6 (grand-mother* or grandmother*)))	47
S4	TI ((daughter* N6 (grand-mother* or grandmother*))) OR AB ((daughter* N6 (grand-mother* or grandmother*))) OR KW ((daughter* N6 (grand-mother* or grandmother*)))	6
S5	TI (((grand-daughter* or granddaughter* or grandaughter*) N6 (grand-mother* or grandmother*))) OR AB (((grand-daughter* or granddaughter* or grandaughter*) N6 (grand-mother* or grandmother*))) OR KW (((grand-daughter* or granddaughter* or grandaughter*) N6 (grand-mother* or grandmother*)))	3
S6	TI (((two or three or "2" or "3" or triad*) N2 generation*)) OR AB (((two or three or "2" or "3" or triad*) N2 generation*)) OR KW (((two or three or "2" or "3" or triad*) N2 generation*))	459
S7	TI (((family or familial) N6 (pair* or triad*))) OR AB (((family or familial) N6 (pair* or triad*))) OR KW (((family or familial) N6 (pair* or triad*)))	66
S8	TI (((mother* or daughter* or grand-mother* or grandmother* or grand-daughter* or granddaughter* or grandaughter*) N3 (pair* or triad*))) OR AB (((mother* or daughter* or grand-mother* or grandmother* or grand-daughter* or granddaughter* or grandaughter*) N3 (pair* or triad*))) OR KW (((mother* or daughter* or grand-mother* or grandmother* or grand-daughter* or granddaughter* or grandaughter*) N3 (pair* or triad*)))	194
S9	TI ((multi-generation* or multigeneration* or inter-generation* or intergeneration* or bi-generation* or bigeneration* or tri-generation* or trigeneration*)) OR AB ((multi-generation* or multigeneration* or inter-generation* or intergeneration* or bi-generation* or bigeneration* or tri-generation* or trigeneration*)) OR KW ((multi-generation* or multigeneration* or inter-generation* or intergeneration* or bi-generation* or bigeneration* or tri-generation* or trigeneration*))	571
S10	S1 OR S2 OR S3 OR S4 OR S5 OR S6 OR S7 OR S8 OR S9	1,697
S11	DE "BONE density"	3,889
S12	DE "OSTEOPOROSIS"	2,898
S13	DE "BONE fractures" OR DE "ANKLE fractures" OR DE "AVULSION fractures" OR DE "CARTILAGE fractures" OR DE "COMMINUTED fractures" OR DE "FACIAL bone fractures" OR DE "FRACTURE healing" OR DE "HEEL bone fractures" OR DE "JONES fracture" OR DE "PELVIC fractures" OR DE "STRESS fractures (Orthopedics)" OR DE "WRIST fractures"	8,283
S14	TI ((bone N3 (densit* or health or mass or mineral* or content* or fracture* or characteristic* or qualit*))) OR AB ((bone N3 (densit* or health or mass or	10,999

	mineral* or content* or fracture* or characteristic* or qualit*))) OR KW ((bone N3 (densit* or health or mass or mineral* or content* or fracture* or characteristic* or qualit*)))	
S15	TI bmd OR AB bmd OR KW bmd	2,450
S16	TI ((osteoporosis or osteopenia)) OR AB ((osteoporosis or osteopenia)) OR KW ((osteoporosis or osteopenia))	4,987
S17	TI ((fracture* N2 (risk* or history or bone*))) OR AB ((fracture* N2 (risk* or history or bone*))) OR KW ((fracture* N2 (risk* or history or bone*)))	3,339
S18	TI (dual-energy N2 absorptiometry) OR AB (dual-energy N2 absorptiometry) OR KW (dual-energy N2 absorptiometry)	2,585
S19	TI ((DXA or DEXA or FRAX)) OR AB ((DXA or DEXA or FRAX)) OR KW ((DXA or DEXA or FRAX))	2,153
S20	S11 OR S12 OR S13 OR S14 OR S15 OR S16 OR S17 OR S18 OR S19	23,057
S21	S10 AND S20	33

SCOPUS
May 9, 2021

Table S7: Scopus Search Strategy:
(((TITLE-ABS-KEY ((familial W/3 (correlation* OR resemblance* OR pattern* OR characteristic* OR similarit* OR component* OR lineage))) OR TITLE-ABS-KEY ((mother* W/6 daughter*)) OR TITLE-ABS-KEY ((mother* W/6 (grand-mother* OR grandmother*))) OR TITLE-ABS-KEY ((daughter* W/6 (grand-mother* OR grandmother*))) OR TITLE-ABS-KEY (((grand-daughter* OR granddaughter* OR grandaughter*) W/6 (grand-mother* OR grandmother*))))) OR ((TITLE-ABS-KEY (((two OR three OR "2" OR "3" OR triad*) W/2 generation*)) OR TITLE-ABS-KEY (((family OR familial) W/6 (pair* OR triad*))) OR TITLE-ABS-KEY (((mother* OR daughter* OR grand-mother* OR grandmother* OR grand-daughter* OR granddaughter* OR grandaughter*) W/3 (pair* OR triad*))) OR TITLE-ABS-KEY ((multi-generation* OR multigeneration* OR inter-generation* OR intergeneration* OR bi-generation* OR bigeneration* OR tri-generation* OR trigeneration*))))) AND ((TITLE-ABS-KEY ((bone W/3 (densit* OR health OR mass OR mineral* OR content* OR fracture* OR characteristic* OR qualit*)) .) OR TITLE-ABS-KEY ((osteoporosis OR osteopenia)) OR TITLE-ABS-KEY ((fracture* W/2 (risk* OR history OR bone*))) OR TITLE-ABS-KEY ((dual-energy W/2 absorptiometry)) OR TITLE-ABS-KEY ((dxa OR dexa OR frax OR bmd))))

543 results

Supplementary 3: ScR Grey Literature Conference Search

Table S8: Grey Literature Conference Search

Conference	Journal	Years	Found	Unique ?
World Congress on Osteoporosis, Osteoarthritis And Musculoskeletal Diseases	Osteoporosis International	2020-2014, 2010	0	—
IOF Regionals: Asia-Pacific Osteoporosis Conference	Osteoporosis International	2019, 2016, 2014-2010	1 (2010)	No
Osteoporosis Conference	Osteoporosis International	2018, 2016, 2014, 2010	0	—
Interdisciplinary Symposium on Osteoporosis	Osteoporosis International	2014, 2013	0	—
Skeletal Health	Osteoporosis International	2014	0	—
European Congress on Osteoporosis and Osteoarthritis	Osteoporosis International	2013, 2012.	1 (2012)	No
Bone Quality Seminar	Osteoporosis International	2013, 2012, 2012	0	—
International Symposium on Osteoporosis	Osteoporosis International	2012, 2011	0	—
Osteoporosis and Bone Conference	Osteoporosis International	2012	1 (2012)	No
IOF Regionals: Middle East & Africa Osteoporosis Meeting	Osteoporosis International	2015, 2012, 2011.	0	—
Annual Meeting of the American Society for Bone and Mineral Research	Journal of Bone and Mineral Research	2020-2010	2 (2014 & 2018)	No

International Conference on Progress in Bone and Mineral Research	Bone	2012	0	—
ECTS Annual Congress	Bone	2011, 2010.	0	—
International Conference on Osteoporosis and Bone Research	Bone	2010, 2009.	0	—
European Symposium on Calcified Tissues	Bone	2010	0	—
IBMS Davos Workshops: Bone Biology & Therapeutics	Bone	2010	0	—
International Conference on Cancer-Induced Bone Disease	Bone	2010	0	—

Supplementary 4: ROI & Radius

Table S9: Categorization and Definition of Regions of Interest (ROIs).

Category	ROIs	Notes/Definitions
Whole Body	Whole Body (WB)	•
Forearm	Forearm (Fore)	•
Radius	Ultradistal Radius (UDRad)	• ~ 4% from distal end of radius<br>• 9mm starting proximally at 9.5mm from joint margin reference line for distal radius [HR-pQCT] [2]
	Mid-Distal Radius (MDRad)	• ~10-30% from distal end of radius<br>• Where there is 5 or 8mm between radius and ulna
	Mid Radius (MRad)	• 2/3 distance from olecranon→ styloid process [3]<br>• Starting proximally at 30% up from wrist [4] [pQCT]<br>• Mid-radius/radial diaphysis
Lumbar Spine	L1-4 (LS)	•
	L2-4	•
Hip	Total Hip (TH)	•
	Femoral Neck (FN)	•
	Trochanter (Troch)	•
	Intertrochanter (iTroch)	•
	Ward's Triangle/Area (Ward)	•
Femur	Femoral Diaphysis/Midshaft	• 50 mm distal from lesser trochanter [5]<br>• Femoral midshaft [6]
Tibia	Proximal Tibia (Prox)	• at 60% of lower leg up from lateral malleolus [7] [pQCT]
	Distal Tibia (Dist)	• Starting 22.5mm from ankle joint [8]<br>• 9mm starting proximally at 22.5mm from joint margin reference line for distal tibia [2]
Heel	Heel/Calcaneus	

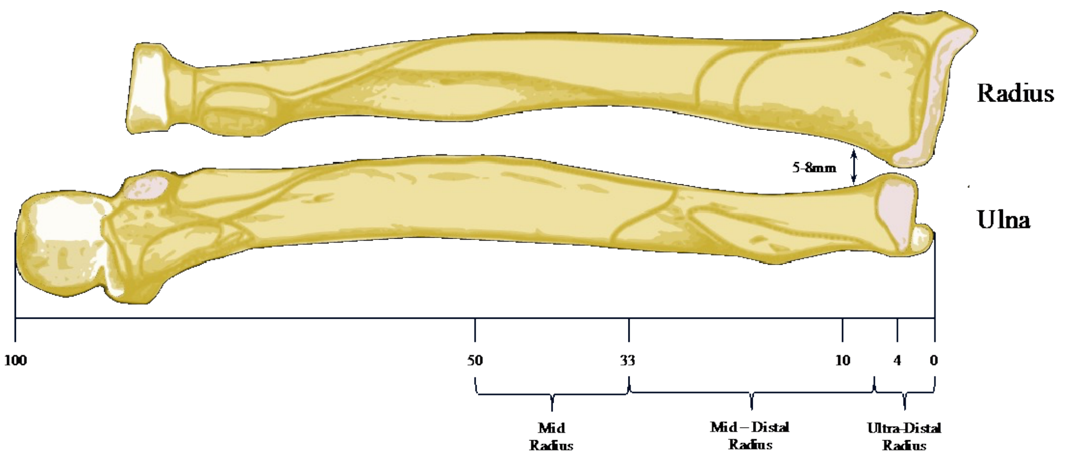

Figure S1: Radial ROI Categorizations. Adapted from: Originally by Henry Vandyke Carter (1831–1897), vectorized and reworked by User:jppialasse, CC BY-SA 4.0, via Wikimedia Commons, with ROI locales [9]

Supplementary 5: Ratios Tables

Note: some studies reported results for multiple regions of interest ROIs within one ROI category, therefore ratio numbers may not directly match number of references.

Reference numbers are not the same as in the publication. See page 19-20 for relevant supplemental references.

Table S10: Ratios of significant: non-significant (: unknown significance) difference (t-test and ANOVA) study-site results reported by included studies per ROI category and daughter-mother pairing.

	**Daughter – Mother Pairings**					**Triad**	**Daughter – Grandmother Pairing**
	**Adolescent – Pre-Menopausal**	**Pre-Menopausal – Pre-Menopausal**	**Adolescent – Mixed-Menopausal**	**Pre-Menopausal – Mixed-Menopausal**	**Pre-Menopausal – Post-Menopausal**	**Adolescent – Pre-Menopausal – Post-Menopausal**	**Adolescent – Post-Menopausal**
**Whole Body**					1[10]:0		
**Radius**	3[3, 4]:0		0:3[2]		3[4, 11]:0		1[4]:0
**Lumbar Spine**	1[5]:1[12]	0:1[13]	1[5]:1[2]	1[13]:0	3[13-15]:1[16]	1[17]:0	

**Hip**	4[5, 18]:2[5, 18]	2[13]:1[13]	5[2, 5]:1 [5]	3[13]:0	7[11, 13-15, 18]:5[11, 13, 14, 16]		2[18]:0
**Femur**	0:1[5]		0:1[5]				
**Tibia**	2[4, 7]:0		1[2]:0		3[4, 7, 11]:0		1[4]:0

Table S11: Ratios of significant: non-significant (: unknown significance) correlation and regression study-site results reported by included studies per ROI category and daughter-mother pairing.

	**Adolescent – Pre-Menopausal**	**Pre-Menopausal – Pre-Menopausal**	**Adolescent – Mixed-Menopausal**	**Pre-Menopausal – Mixed-Menopausal**	**Mixed-Menopausal – Mixed-Menopausal**	**Pre-Menopausal – Post-Menopausal**	**Mixed-Menopausal – Post-Menopausal**	**Post-Menopausal – Post-Menopausal**	**Adolescent – Post-Menopausal (D-G)**
**Whole Body**	2[19, 20]:1[21]	-	-	-	-	1[22]:1[10]	-	-	-
**Forearm**	-	-	1[23]:0	-	-	2[24]:0	-	-	-
**Radius**	2[3]:0:*2[25]*	-	-	-	0:1[26]	4[8]:2[22, 26]	-	-	-

**Lumbar Spine**	3[12, 17, 21]:0:*1[25]*	2[13, 27]:0	1[5]:0	1[13]:0	-	7[6, 8, 16, 24, 28, 29]:3[13, 17, 22]	1[28]:0	0:1[28]	0:1[17]
**Hip**	1[21]:1[21]:2[25]	2[13]:1[13]	2[5]:2[5]	3[13]:0	1[30]:0	14[6, 8, 13, 16, 24, 28, 29]:5[13, 22, 29]	0:2[28]	1[28]:1[28]	-
**Femur**	0:0:*1[25]*	-	1[5]:0	-	-	0:1[6]	-	-	-
**Tibia**	-	-	-	-	-	1[8]:0	-	-	-
**Heel**	-	-	-	-	-	3[22, 28, 31]:1[28]	0:2[28]	2[28]:0	-

Table S12: Ratios of significant: non-significant (: unknown significance) heritability study-site results reported by included studies per ROI category and daughter-mother pairing.

	**Adolescent – Pre-Menopausal**	**Adolescent – Mixed-Menopausal**	**Mixed-Menopausal – Mixed-Menopausal**	**Adolescent – Post-Menopausal**	**Pre-Menopausal – Post-Menopausal**	**Post-Menopausal – Post-Menopausal**

**Whole Body**	1[21]:0					
**Radius**	4[2, 3]:1[2]	3[2]:0		2[2]:1[2]		
**Lumbar Spine**	3[2, 12, 21]:0	1[2]:0		1[2]:0	2[28, 29]:0	0:1[28]
**Hip**	4[2, 21]:0	2[2]:0	0:0:*1[30]*	2[2]:0	2[28]:4[29]	1[28]:1[28]
**Tibia**	1[2]:0	1[2]:0		1[2]:0		
**Heel**					1[28]:1[28]	2[28]:0

References

1. Tricco, A.C., et al., *PRISMA extension for scoping reviews (PRISMA-ScR): Checklist and explanation.* Annals of Internal Medicine, 2018. **169**(7): p. 467-473.
2. Pepe, J., et al., *Within- and across-sex inheritance of bone microarchitecture.* The Journal of Clinical Endocrinology & Metabolism, 2017. **102**(1): p. 40-45.
3. Tylavsky, F.A., et al., *Familial resemblance of radial bone mass between premenopausal mothers and their college-age daughters.* Calcified Tissue International, 1989. **45**(5): p. 265-72.
4. Wang, Q., et al., *Is bone loss the reversal of bone accrual? Evidence from a cross-sectional study in daughter-mother-grandmother trios.* Journal of Bone and Mineral Research, 2011. **26**(5): p. 934-40.
5. Henderson, N.K., et al., *Bone density in young women is associated with body weight and muscle strength but not dietary intakes.* Journal of Bone and Mineral Research, 1995. **10**(3): p. 384-93.
6. Seeman, E., et al., *Reduced bone mass in daughters of women with osteoporosis.* New England Journal of Medicine, 1989. **320**(9): p. 554-8.
7. Xu, L., et al., *Fat mass accumulation compromises bone adaptation to load in Finnish women: A cross-sectional study spanning three generations.* Journal of Bone and Mineral Research, 2010. **25**(11): p. 2341-9.
8. Nagy, H., et al., *Impaired trabecular and cortical microarchitecture in daughters of women with osteoporotic fracture: The MODAM study.* Osteoporosis International, 2013. **24**(6): p. 1881-9.
9. Bonnick, S.L. and L.A. Lewis, *Bone densitometry for technologists*. 3rd 2013. ed. 2013, New York, NY: Springer New York.
10. Ulrich, C.M., et al., *Bone mineral density in mother-daughter pairs: Relations to lifetime exercise, lifetime milk consumption, and calcium supplements.* The American Journal of Clinical Nutrition, 1996. **63**(1): p. 72-9.
11. Nagy, H., et al., *Family resemblance of bone turnover rate in mothers and daughters: The MODAM study.* Osteoporosis International, 2015. **26**(3): p. 921-30.
12. Francois, S., et al., *Heritability of bone mineral density.* Revue du Rhumatisme (English ed.), 1999. **66**(3): p. 146-51.
13. Lutz, J. and R. Tesar, *Mother-daughter pairs: Spinal and femoral bone densities and dietary intakes.* The American Journal of Clinical Nutrition, 1990. **52**(5): p. 872-7.
14. Lee, S.Y., et al., *Age-related bone loss is associated with FGF21 but not IGFBP1 in healthy adults.* Experimental Physiology, 2020. **105**(4): p. 622-631.
15. Shetty, S., et al., *Bone turnover markers and bone mineral density in healthy mother-daughter pairs from South India.* Clinical Endocrinology, 2016. **85**(5): p. 725-732.
16. McKay, H.A., et al., *Familial comparison of bone mineral density at the proximal femur and lumbar spine.* Bone and Mineral, 1994. **24**(2): p. 95-107.
17. Ohta, H., et al., *Familial correlation of bone mineral density, birth data and lifestyle factors among adolescent daughters, mothers and grandmothers.* Journal of Bone & Mineral Metabolism, 2010. **28**(6): p. 690-5.

18. Wang, Q., et al., *Growth and aging of proximal femoral bone: A study with women spanning three generations.* Journal of Bone and Mineral Research, 2015. **30**(3): p. 528-34.
19. Cheng, S., et al., *Trait-specific tracking and determinants of body composition: A 7-year follow-up study of pubertal growth in girls.* BMC Medicine, 2009. **7**(101190723): p. 5.
20. Jouanny, P., et al., *Environmental and genetic factors affecting bone mass. Similarity of bone density among members of healthy families.* Arthritis and Rheumatism, 1995. **38**(1): p. 61-7.
21. Nabulsi, M., et al., *Gender differences in the heritability of musculoskeletal and body composition parameters in mother-daughter and mother-son pairs.* Journal of Clinical Densitometry, 2013. **16**(2): p. 223-30.
22. Krall, E.A. and B. Dawson-Hughes, *Heritable and life-style determinants of bone mineral density.* Journal of Bone and Mineral Research, 1993. **8**(1): p. 1-9.
23. Magarey, A.M., et al., *Familial and environmental influences on bone growth from 11-17 years.* Acta Paediatrica, 1999. **88**(11): p. 1204-10.
24. Hansen, M.A., et al., *Is heritability a risk factor for postmenopausal osteoporosis?* Journal of Bone and Mineral Research, 1992. **7**(9): p. 1037-43.
25. Ferrari, S.L., et al., *Childhood fractures are associated with decreased bone mass gain during puberty: An early marker of persistent bone fragility?* Journal of Bone and Mineral Research, 2006. **21**(4): p. 501-7.
26. Sowers, M.R., et al., *Familial resemblance of bone mass in adult women.* Genetic Epidemiology, 1986. **3**(2): p. 85-93.
27. Picard, D., et al., *Familial resemblance of bone mineral density between females 18 years and older and their mothers.* Canadian Journal of Public Health. Revue Canadienne de Sante Publique, 2001. **92**(5): p. 353-8.
28. Danielson, M.E., et al., *Familial resemblance of bone mineral density (BMD) and calcaneal ultrasound attenuation: The BMD in mothers and daughters study.* Journal of Bone and Mineral Research, 1999. **14**(1): p. 102-10.
29. Runyan, S.M., et al., *Familial resemblance of bone mineralization, calcium intake, and physical activity in early-adolescent daughters, their mothers, and maternal grandmothers.* Journal of the American Dietetic Association, 2003. **103**(10): p. 1320-5.
30. Blain, H., et al., *Lean mass plays a gender-specific role in familial resemblance for femoral neck bone mineral density in adult subjects.* Osteoporosis International, 2006. **17**(6): p. 897-907.
31. Drozdzowska, B. and W. Pluskiewicz, *Quantitative ultrasound at the calcaneus in premenopausal women and their postmenopausal mothers.* Bone, 2001. **29**(1): p. 79-83.
